# In situ buccal carcinoma in a teenager after hematopoietic stem cell transplantation: A case report

**DOI:** 10.1097/MD.0000000000022781

**Published:** 2020-10-23

**Authors:** Yanbin Liu, Wei Yuan, Man Li, Long Cheng, Jinsuo Yang, Boya Yin, Xin Huang

**Affiliations:** Department of Oral and Maxillofacial-Head and Neck Oncology, Beijing Stomatological Hospital, School of Stomatology, Capital Medical University, Beijing, China.

**Keywords:** graft-versus-host disease, hematopoietic stem cell transplantation, in situ buccal carcinoma

## Abstract

**Rationale:**

Hematopoietic stem cell transplantation (HSCT) is the most effective treatment for the majority of patients who have malignant haemolytic disease. Although the success rate of HSCT has increased, the increasing number of cases suffering from secondary solid malignancies after HSCT has attracted more interest recently.

**Patient concerns:**

A 16-year-old female patient from China presented with a crusty and painful lesion on the left buccal mucosa with a history of chronic graft-versus-host disease following allogeneic HSCT for acute myeloid leukaemia.

**Diagnosis:**

An incisional biopsy of the lesion showed stratified squamous epithelium mucosa with severe dysplasia (carcinoma in situ). Subsequently, a wide local excision was performed and histological examination revealed early infiltrating squamous epithelial mucosa (carcinoma in situ).

**Interventions:**

She was being treated in the oral and maxillofacial surgery clinic with an incisional biopsy of the left buccal mucosa. She also received a wide local excision.

**Outcomes:**

Follow-up for 4 years showed no recurrence.

**Lessons:**

This case helps raise awareness of the diagnosis of oral symptoms in young patients after HSCT. Due to the increasing application of HSCT, raising awareness in oral and dental physicians may be required to improve long-term clinical outcome of patients who underwent HSCT.

## 1 Introduction

Hematopoietic stem cell transplantation (HSCT) is a vital therapeutic method for most patients who have malignant hematological disorders. Graft-versus-host disease (GVHD) usually occurs after allogeneic HSCT, which can appear on different sites such as oral mucosa, liver, and skin and are related to a high risk of secondary solid malignancy. According to previous reports, secondary solid malignancies account for approximately 5% to 10% of late death in patients after HSCT,^[1–5]^ although the occurrence of head and neck squamous cell carcinoma (SCC) is uncommon. In this report, we describe a case of in situ buccal carcinoma in a teenager who developed chronic GVHD following allogeneic HSCT for acute myeloid leukemia.

## 2 Case presentation

In February 2016, a 16-year-old female was referred to the oral and maxillofacial surgery clinic at the Beijing Stomatological Hospital with a chief complaint of a crusty and painful lesion on the left buccal mucosa. She was previously diagnosed with acute myeloid leukemia in 2008. In January 2014, she underwent an allogeneic hematopoietic stem cell transplantation (HSCT) after a course of induction chemotherapy. After transplantation, long-term oral cyclosporine was taken. She had a history of skin and oral chronic GVHD, which was treated with methotrexate and cyclosporine and gradually subsided 2 years later. She did not report any oral symptoms until her first hospital visit.

**Figure 1:**
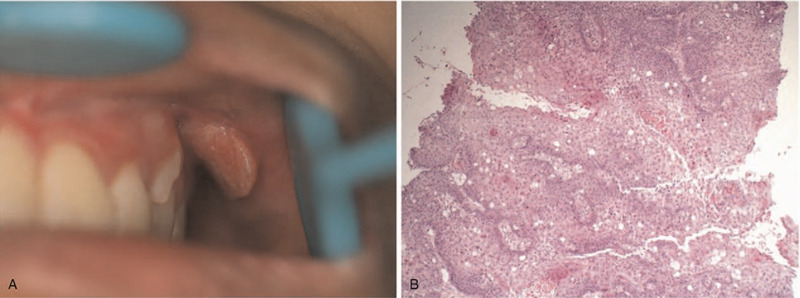
(A) Oral lesions on the left buccal mucosa. (B) Histopathologic view of lesion showing severe hyperplasia of the squamous epithelial mucosa (carcinoma in situ). Haematoxylin and eosin stain. 100× magnification.

**Figure 2:**
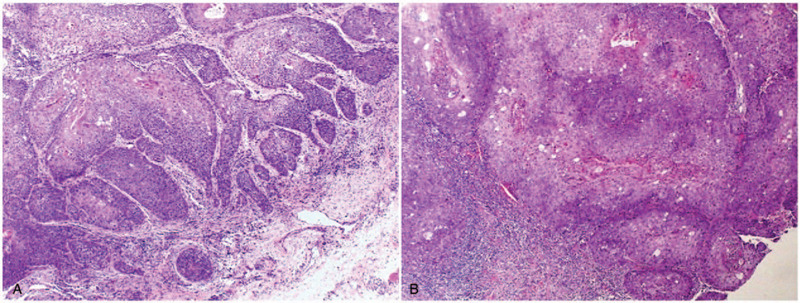
(A) Intraoperative and (B) postoperative histopathologic examination. Microscopic image showing early infiltrating squamous epithelial mucosa (carcinoma in situ). Haematoxylin and eosin stain. 100× magnification.

**Figure 3:**
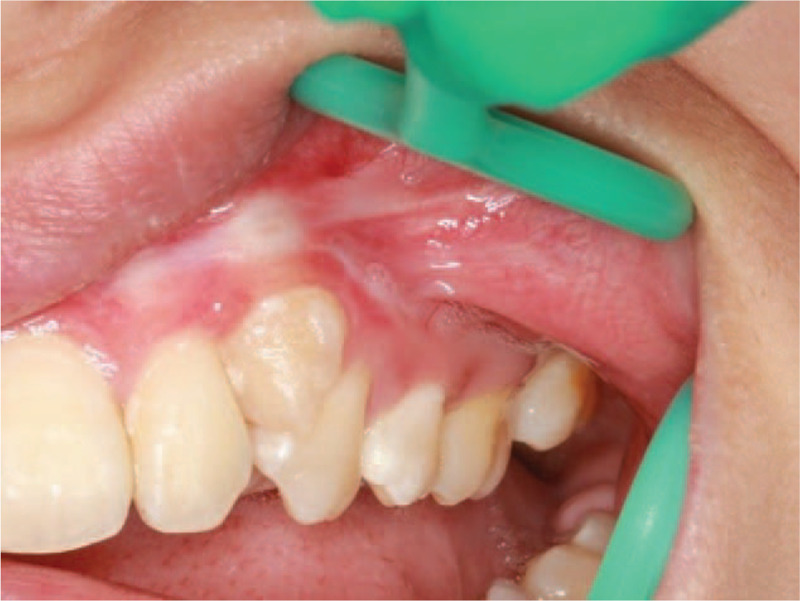
The left buccal region after surgery.

**Figure 4:**
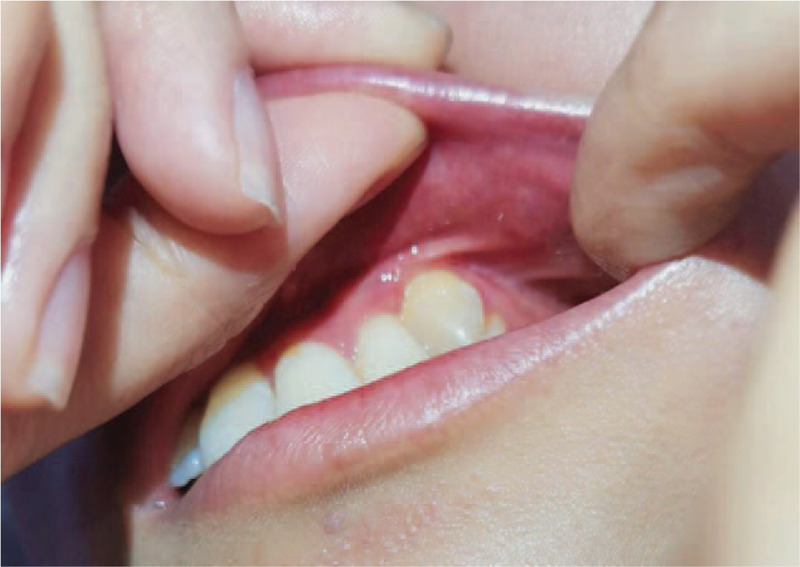
Patient's self-taken image of the left buccal region after surgery (March 2020).

The patient had normal adolescent development and further examination did not reveal any remarkable body features or abnormal findings of the cervical lymph nodes. There was no obvious asymmetry in the patient's face. Intraoral examination revealed a raised pink neoplasm with clear demarcation (1.3 × 1.2 cm) on the left buccal mucosa. The surface of the neoplasm was slightly uneven and partially white and the marginal tissue was soft with pressure pain (Fig. 1A). An incisional biopsy of the lesion showed stratified squamous epithelium mucosa with severe dysplasia (carcinoma in situ; Fig. 1B). Based on these findings, the malignancy was staged at TisN0M0 and she subsequently underwent a wide local excision. Intraoperative and postoperative histopathologic examinations confirmed early infiltrating squamous epithelial mucosa (carcinoma in situ; Fig. 2) and we did not detect human papillomavirus (HPV) DNA. The tumor did not recur at a follow-up examination after 17 months (Fig. 3) and 4 years (Fig. 4).

Ethics approval was obtained from the Ethics Committee of the Beijing Stomatological Hospital. The patient has provided informed consent for publication of the case. Informed written consent was obtained from the patient's parents for publication of this case report and accompanying images.

## 3 Discussion

HSCT is broadly used as a potentially therapeutic treatment for patients with various malignant hematological disorders, bone marrow failure disease, and congenital immune deficiencies.^[6]^ The number of allogeneic HSCT has increased steadily over the past decades worldwide.^[7–9]^ To date, advances in transplant medicine has greatly improved the patient's long-term survival rate. With improved clinical outcomes after HSCT, there is rising interest in secondary solid tumors in long-term survivors. Previously published reports suggest that risk factors for developing solid tumors include treatment with radiation, chronic GVHD, male gender, younger age, and immune-suppressive therapy. These factors also increased the relative risk of oral squamous cell carcinoma.^[10–13]^ The majority of solid malignancies following allogeneic HSCT are SCC affecting skin, genitourinary tract, and oral cavity.^[14]^ Rizzo et al analyzed 28,874 allogeneic HSCT patients and reported that people with GVHD have 5 times the incidence of SCC compared with the general population.^[10]^ In the past, some investigators suggested that patients with Fanconi anemia have a higher risk of head and neck SCC with poor survival.^[15]^

Our report of the current case of carcinoma in situ following HSCT that occurred in buccal mucosa has not yet been fully understood. In particular, we need to elucidate the exact pathogenic mechanism of the development of oral SCC in patients with HSCT and history of GVHD that was controlled by drug treatment. Although the source of the patient's carcinoma in situ is unknown, it may be the result of multifactorial interactions, including genetic predisposition, individual behaviors, long-terms effects of chemotherapeutic drugs and radiation, and viral infections.^[14]^

In the cases reported by Budrukkar et al, the authors realised that tobacco or alcohol misuse may hasten the occurrence of head and neck SCC. In addition, these advanced tumors progress rapidly and occur early on with multiple recurrences, implying that head and neck SCC in these patients are especially aggressive.^[16]^ However, we describe a case of an unusual in situ carcinoma of the buccal mucosa that developed in a teenager without history of smoking and drinking. Kata et al presented 2 p16 HPV-positive patients with oral SCCs after HSCT, suggesting the importance of screening and early intervention for HSCT-related oral lesions.^[17]^ Nonetheless, we did not detect HPV DNA in our patient. Gallagher et al evaluated 926 patients who underwent HSCT between 1985 and 2003. They found that patients who received allogeneic HSCT at older age and those who accepted female transplants have an heightened incidence of developing secondary solid cancers.^[18]^ It was notable that our patient had a history of HSCT from an unrelated matched donor graft at 14 years old. Curtis et al examined in 19,229 patients who received transplants and described the cumulative occurrence rate of new solid cancers of 2.2% at 10 years and 6.7% at 15 years.^[5]^ The risk of secondary solid tumors begins to increase around 10 years after transplantation and continues even 20 years later.^[5]^ However, our patient reported oral symptoms just 2 years after transplantation.

Given the patient's history of allogeneic HSCT and her young age, an exact clinical diagnosis was challenging. Pseudoepitheliomatous hyperplasia (PEH) is a type of epithelial hyperplasia that is often associated with chronic inflammation, trauma, granular cell tumors, and chronic irritation.^[19]^ It resembles well-differentiated SCC. Epithelial malignancies in young patients are unexpected and thus, pathologists find it difficult to differentiate between PEH and SCC in children and adolescents.^[20]^ Hence, a diagnosis of SCC should be distinguished carefully from PEH, particularly among young patients.

## 4 Conclusions

In conclusion, the diagnosis of in situ carcinoma of buccal mucosa in a young patient just 2 years after allogeneic HSCT was rare. Carcinoma in situ is typically treated by simple excision. The possibility of tumor recurrence cannot be completely ruled out because the young age of the patient developing SCC of the head and neck after GVHD has not been reported before. Ultimately, regular follow-up is necessary after removal of oral neoplasms. Therefore, heightened vigilance by oral and dental physicians may be required for patients after HSCT. Further research into the molecular mechanisms are required to completely comprehend this rare disease.

## Author contributions

**Data curation:** Long Cheng, Jinsuo Yang, Boya Yin.

**Resources:** Yanbin Liu, Man Li.

**Supervision:** Xin Huang.

**Writing – original draft:** Yanbin Liu.

**Writing – review & editing:** Xin Huang, Wei Yuan.
